# Acute basophilic leukaemia in a three-month-old calf

**DOI:** 10.1186/s13028-015-0141-z

**Published:** 2015-09-03

**Authors:** Eva-Maria Laabs, Reinhard Mischke, Peter Dziallas, Arianna Maiolini, Andrea Tipold, Barbara Raddatz, Christina Puff, Jürgen Rehage

**Affiliations:** Clinic for Cattle, University of Veterinary Medicine Hannover Foundation, Bischofsholer Damm 15, 30173 Hannover, Germany; Department of Small Animal Medicine and Surgery, University of Veterinary Medicine Hannover Foundation, Bünteweg 9, 30559 Hannover, Germany; Department of Pathology, University of Veterinary Medicine Hannover Foundation, Bünteweg 17, 30559 Hannover, Germany

**Keywords:** Cattle, Paresis, Myeloid leukaemia, Thrombocytopenia, Haemorrhagic disorder

## Abstract

A three-month-old female Holstein–Friesian calf was presented with acute tetraparesis. After neurological examination a multifocal lesion in the central nervous system was suspected with the most pronounced lesions between the third thoracic and the third lumbar vertebrae. Haematological examination revealed moderate anaemia as well as severe thrombocytopenia, neutropenia and leucocytosis. A blood smear and bone marrow aspirate exhibited predominantly blasts with basophilic granulation leading to a diagnosis of acute (myeloid) leukaemia with involvement of the basophilic lineage or an acute basophilic leukaemia. Magnetic resonance imaging revealed spinal cord compression; at necropsy, extensive localised haemorrhages extending into the thoracic vertebral canal were found. Histopathology revealed a large population of blast cells in several tissues including the meninges. Due to multifocal detection of neoplastic cells in the vascular system, neoplasia of the haematopoietic system was assumed in agreement with haematological findings. Signs of paresis could be explained by intramedullary spinal cord haemorrhage and myeloid infiltrations of meningeal vessels. In conclusion, despite its rarity, acute myeloid leukaemia with involvement of the basophilic lineage may be considered in diagnosing calves with progressive deteriorating general condition, paresis, leucocytosis with moderate basophilic differentiation or haemorrhagic disorders.

## Background

Leukaemia is a malignant disease of any cellular element in the peripheral blood or bone marrow i.e., including red blood cells and platelets. Neoplastic cells are found in the bone marrow associated with displacement of normal haematopoietic cells and, in most cases, also in peripheral blood. Depending on the progression, maturity level, and origin of the cells involved, acute or chronic leukaemia is differentiated [1]. According to the French-American-British-classification for human leukaemia, acute leukaemia may be divided into two groups, lymphoblastic and myeloid [2]. Similarly, a classification of myeloid neoplasms together with acute leukaemia was published by the World Health Organisation (WHO) in 1999 [3] and in 2009 [4].

Acute myeloid leukaemia (AML) is a rare condition in humans that constitutes approximately 20 % of childhood leukaemia cases [5]. In dogs, many cases of AML, including basophilic leukaemia [6–8], have been reported [9–11]. Contrarily, myeloproliferative diseases in large animals are rare, with few reported cases in horses [12]. In cattle, especially non-lymphocytic leukaemia is uncommon, whereby reports comprise of: acute myelomonocytic leukaemia [13], acute myeloblastic leukaemia [14], monocytic leukaemia [15], mast cell leukaemia [16] and basophilic leukaemia [17].

This report describes clinical-, haematological-, magnetic resonance imaging (MRI)- and pathological findings of a calf suffering from AML with involvement of the basophilic lineage or an acute basophilic leukaemia.

## Case presentation

### History

A three-month-old female Holstein–Friesian calf weighing 84 kg was admitted to the Clinic for Cattle of the University of Veterinary Medicine Hannover Foundation with acute paresis in all four limbs as well as pneumonia and fever. According to the owner, the calf had been susceptible to various diseases since birth, even though colostrum supply was deemed sufficient.

### Clinical findings

**Fig. 1:**
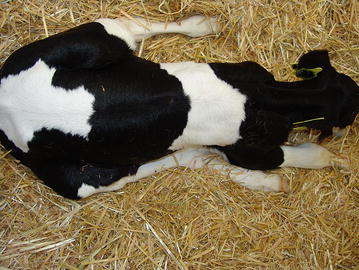
Calf with its hind legs stretched beside its body

On clinical examination the calf was depressed, recumbent, yet in good body condition, and exhibited a good appetite (calf starter and hay). Rectal temperature was within normal limits (38.9 °C), and examination of the cardiovascular system revealed a marginal tachycardia (pulse rate: 100 beats/min), yet no abnormalities. The calf showed an inspiratory dyspnea with an increased respiratory rate of 54 breaths/min and exaggerated expiratory efforts accompanied by occasional spontaneous unproductive cough. Increased respiratory sounds were audible over the entire lung fields upon thoracic auscultation on both sides. Mild sero-mucous nasal discharge was noticed, although nasal and oral mucous membranes were of normal pale-pink colour. Palpable external lymph nodes were unremarkable. In recumbent position, the calf’s hind legs remained stretched alongside its body (Fig. 1). In a standing position supported on both sides, the calf displayed an arched back as well as hyperflexion of the tarsal joints and knuckling over in the fetlock joints of both hind limbs. During movement, the calf exhibited tetraparesis and a generalised ataxia, which was more pronounced in the hind limbs. Vision, other cranial nerve functions, panniculus reflex and spinal reflexes were normal, but skin sensation appeared to be reduced in both hind limbs. During hospitalisation, paresis of the hind limbs worsened in comparison to the day of admission. Other neurologic signs remained unchanged. The most pronounced neurological alteration was therefore suspected to be in the spinal cord between the third thoracic and the third lumbar vertebrae because of the non-ambulatory paraparesis with normal spinal cord reflexes. During the collection of blood samples for laboratory analysis (as described later) prolonged bleeding at injection sites was conspicuous.

### Laboratory findings

Blood samples were collected from the jugular vein, and ethylenediaminetetraacetate (EDTA) tubes were used for a blood smear and haematological examination (Celltac alpha MEK-6450K, Nihon Kohden, Tokyo, Japan). Additional blood was collected into tubes without anticoagulant and after clotting, was centrifuged at 1500*g* for 15 min at 4 °C for harvesting serum. Afterwards, the samples were analysed immediately. At presentation, haematological examination revealed moderate anaemia (red blood cell count 3.93 × 10^12^ erythrocytes/l, reference interval 5.0–10.0 × 10^12^ erythrocytes/l; haemoglobin concentration 62 g/l, reference interval 80–140 g/l; haematocrit 0.21 l/l, reference interval 0.30–0.40 l/l [18]), thrombocytopenia (27 × 10^9^ thrombocytes/l; reference interval 200–800 × 10^9^ thrombocytes/l [18]), and leucocytosis (37.2 × 10^9^ leucocytes/l; reference interval 5.0-12.0 × 10^9^ leucocytes/l [18]). Clinical chemistry revealed neither abnormalities in liver and kidney parameters (total bilirubin, glutamate dehydrogenase, aspartate aminotransferase, gamma-glutamyltransferase, urea, and creatinine) nor in electrolytes. An arterial blood gas analysis revealed partial oxygen pressure and partial carbon dioxide pressure: 84 mmHg (reference interval 63.85 ± 10.82 mmHg [19]) and 42 mmHg (reference interval 45.25 ± 3.69 mmHg [19]), respectively.

While the calf was anaesthetised for MRI (described later), a lumbar cerebrospinal fluid (CSF) sample was collected. Furthermore, bone marrow aspirates from the sternum and tuber coxae (after surgical preparation of the skin) were taken using disposable needles for bone marrow or CSF sampling (19 G, 1.3 × 75 mm; B. Braun, Melsungen, Germany) and collected into potassium EDTA tubes. Specimens were prepared using the squash technique.

**Fig. 2:**
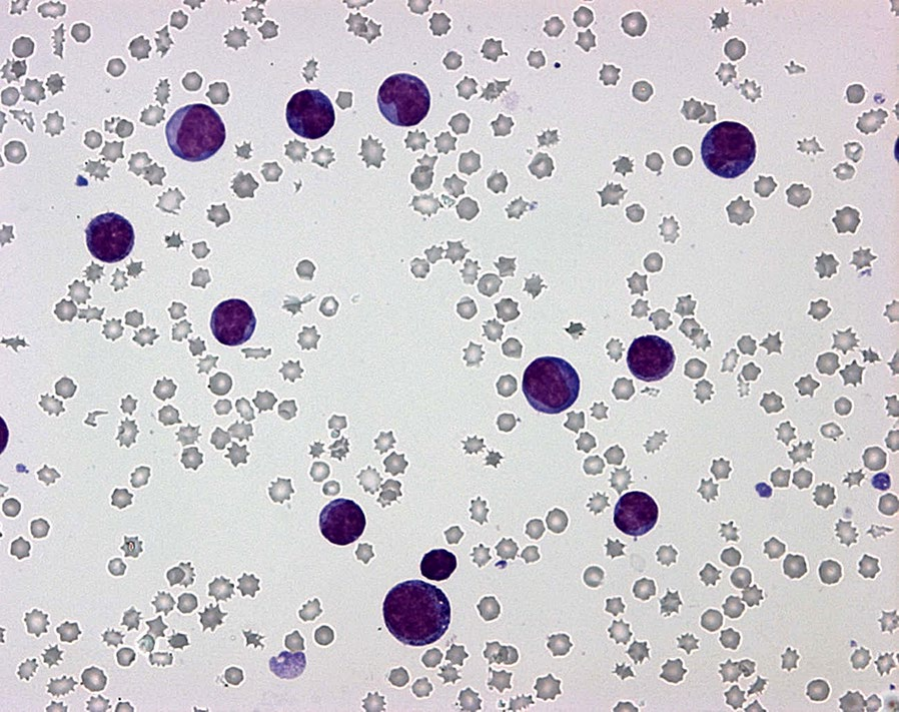
Distinct increase in numbers of white blood cells. Blood smear showing a predominance of polymorphic blasts, some with lobate nucleus and nuclear fragmentation and/or with basophilic granulation (Pappenheim-stain; ×400)

**Fig. 3:**
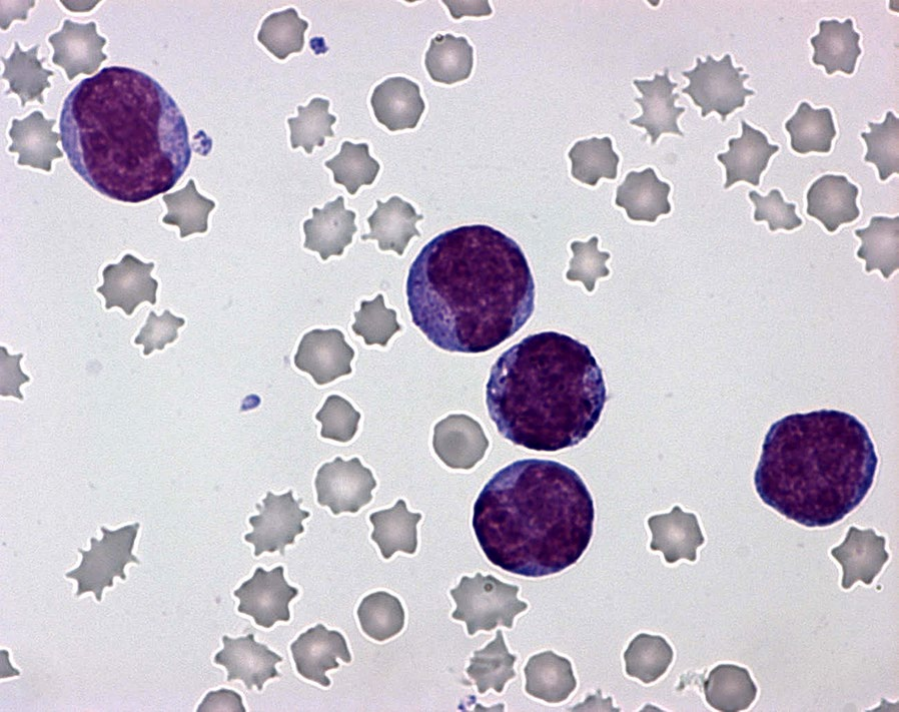
Distinct increase in numbers of white blood cells. Blood smear. The *image* has a higher magnification as Fig. 2 (Pappenheim-stain; ×1000)

**Fig. 4:**
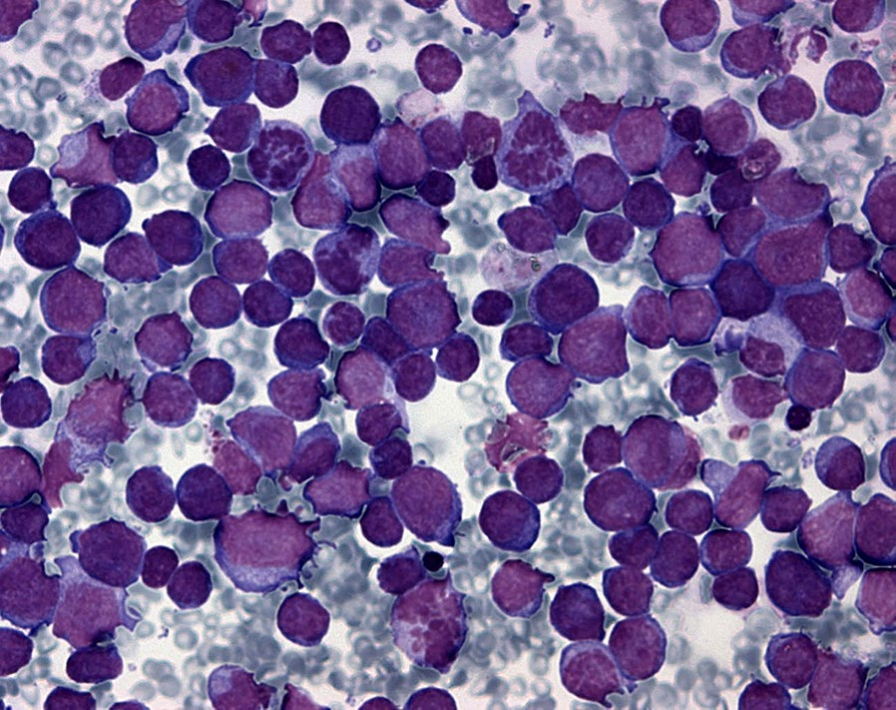
Highly cellular bone marrow aspirate. Bone marrow aspirate. There is high cellularity, a predominance of blasts and minimal residual normal haematopoietic cells. Some cells show distinct basophilic granulation. Findings are consistent with acute basophilic leukaemia (Pappenheim-stain; ×400)

**Fig. 5:**
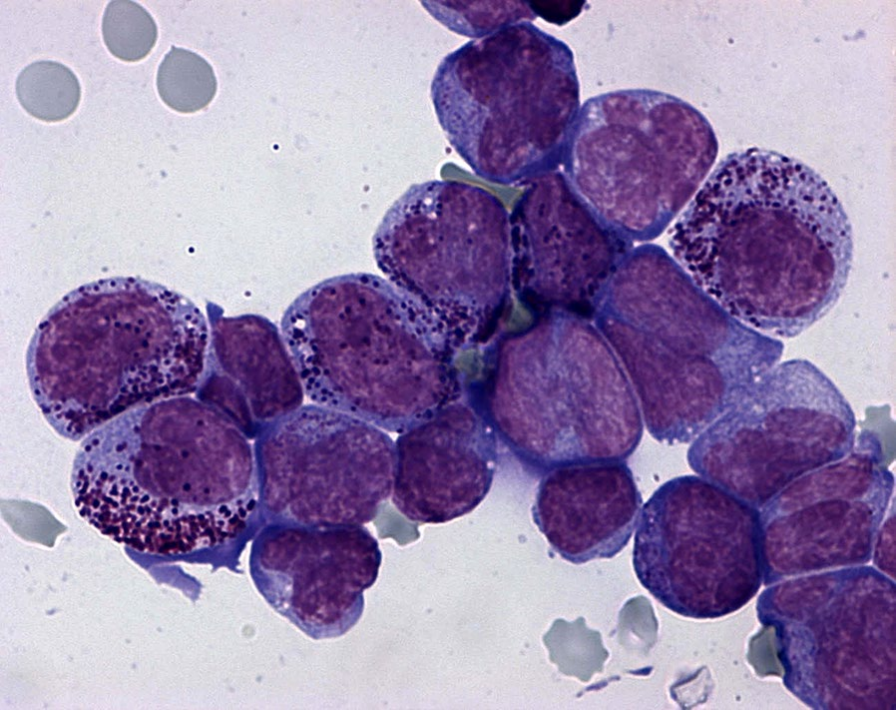
Highly cellular bone marrow aspirate. Bone marrow aspirate. The image has a higher magnification as Fig. 4 (Pappenheim-stain; ×1000)

On Pappenheim staining of the blood smear, (May Grünwald-Giemsa-stain) the nucleated white blood cells comprised 88 % small to medium sized blast cells with diameters mostly between 10 and 14 µm. These cells contained a large oval to round nucleus composed of fine chromatin and possessed several nucleoli as well as a small quantity of basophilic cytoplasm. A little more than one-fifth of the blasts (22 %) showed a more or less distinctly lobed nucleus and some of the blasts showed nuclear fragmentation. In addition, approximately 20 % of the blast cells showed purple granulation, often distinct and partially lying over the nucleus indicating a basophilic origin (Figs. 2, 3). Morphology of approximately 5 % of the cells complies with basophilic myelocytes (coarser chromatin, more and less basophilic cytoplasm, distinct purple granulation), whereas more mature basophilic maturation stages were only occasionally present (<1 %). Lymphocytes (5 %) were reduced and neutrophilic granulocytes (2 %) were almost absent. In addition, four nucleated red blood cells/100 white blood cells were detected. Erythrocytes showed mild anisocytosis, and occasionally basophilic stippling. The examination of the bone marrow aspirate revealed a cell-rich aspirate containing only very few spicules. The cellularity was high, but only occasionally megakaryocytes (on average less than one per low power field i.e. 10× magnification) were present. More than 90 % of the nucleated cells were blast cells of those a small proportion showed a tendency to lobulation and basophilic granulation (Figs. 4, 5). Mitotic figures occurred in high numbers (on average approximately four per high power field, i.e. 400× magnification), whereas the number of precursor cells (mainly normoblasts) of residual normal haematopoietic cells was almost negligible.

The CSF was watery-clear. Protein measured 0.3 g/l (reference interval 0.1–0.4 g/l [20]), and the number of mononuclear leucocytes was 11 × 10^9^ leucocytes/l (reference interval 0–10 × 10^9^ leucocytes/l [20]). However, no neoplastic cells were detected in the CSF.

Considering these findings, a basophilic leukaemia with multifocal involvement of the central nervous system was suspected including a cervical and thoracolumbar lesion. Additionally, a mild form of sub-acute bronchopneumonia was diagnosed.

Further examinations including advanced diagnostic imaging techniques such as MRI were implemented.

### Further clinical diagnostics

#### MRI

**Fig. 6:**
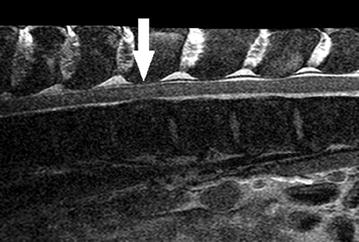
MRI scan of the spine. Slight extramedullary spinal cord compression occurred around the first lumbar vertebra (pointed out by an *arrow*). Ventral disruption of the FLS column due to heterogeneous, hypointense material was visible. The compression started directly above the 13th thoracic vertebra and spanned the entire length of this vertebral body. Due to the size of the calf, a suboptimal coil had to be implemented (Q-Body-Coil), resulting in reduced image quality

Prior to MRI examination, the calf was pre-medicated with xylazine and ketamine [21]. Following intubation, anaesthesia was maintained with isoflurane [21]. The MRI of the thoracic and lumbar spine was conducted with a 3.0 T Philips Achieva MRI scanner (Philips Medical Systems, PC Best, The Netherlands) as described previously [22]. Slight extramedullary spinal cord compression occurred around the first lumbar vertebra. Ventral disruption of the fat-liquor cerebrospinalis (FLS) column due to heterogeneous, hypointense material (Fig. 6) was visible. The compression started directly above the 13th thoracic vertebra and spanned the entire length of this vertebral body. Additionally, multifocal intramedullary lesions occurred in the area of the second to tenth thoracic vertebrae. These lesions were heterogeneous in appearance, either hyperintense or hypointense in comparison to the surrounding tissue of the myelon.

### Clinical diagnosis

The clinical diagnosis was acute basophilic leukaemia with associated hypoplastic anaemia and thrombocytopenia causing a haemorrhagic disorder with multifocal involvement of the central nervous system including suspected alterations in the thoracic/lumbar vertebral column.

Subsequent to the owner’s consent, the calf was euthanised with barbiturates according to manufacturers´ instructions and a postmortem examination was carried out.

### Post mortem findings

At necropsy, the carcass appeared moderately anaemic with multifocal to coalescing ecchymoses (up to 2–3 cm in diameter) and petechial haemorrhages. Few haemorrhages occurred in the oral mucosa, in the parenchyma of the heart and lung, and a moderate number were found in the mucosa of the pharynx, larynx, and oesophagus. A large haematoma was found subcutaneously over the right shoulder joint (30 cm × 10 cm × 20 cm). Furthermore, a severe, acute haemorrhage was found subcutaneously in the medial third of the thorax between the fifth and tenth rib extending partially into the paravertebral musculature, vertebral canal, sternum, and intercostal muscles. Lymph nodes and spleen were enlarged up to twice the original size. With regard to other organs, excepting agonal changes no abnormalities could be detected. Samples for subsequently conducted histopathology were fixed in 10 % neutral-buffered formalin, routinely processed and embedded in paraffin wax. Sections (5 µm) were stained with haematoxylin and eosin. Histopathology revealed a severe infiltration of the popliteal, tracheobronchial and mesenterial lymph nodes with a round, blastoid cell population (20 µm in diameter). This cell population had a low to moderate amount of eosinophilic cytoplasm and an eccentrically located, round to oval, moderately heterochromatin rich nucleus with a basophilic, centrally located, round nucleolus (1–2 µm in diameter). The mitotic rate ranged between zero and six per high power field (400× magnification) depending on the location. Severe infiltration of this blastoid cell population was also detected in vessels and tissues of muscles (triceps brachii muscle, quadriceps femoris muscle and longissimus dorsi muscle), liver, lungs, spleen, and kidney, as well as in the epi- and pericardium and meningeal vessels of the cerebrum and cerebellum. Additionally, bone marrow obtained from the sternum, femoral head, and a rib showed similar cell infiltration with displacement of the original cells.

## Discussion

The diagnosis of acute (blast cell) leukaemia in the presented calf was based on results of the haemogram, bone marrow aspirate and post mortem examination. A high proportion of neoplastic cells within the vascular system as well as their morphology and distributional pattern indicated neoplasia of the haematopoietic system and led to a diagnosis of a myeloid leukaemia with moderate basophilic differentiation. Myeloid leukaemia of basophils was first described in 1906 [23]; however, it was incorporated as a separate neoplasia among the AML as recently as 1999 [3] and 2009 [4] by the WHO. Basophilic leukaemia is a rare disease, accounting for only 5 % of all leukaemia cases in humans [24]. In dogs it is more frequently seen [6–8, 25], whereas it is rare in horses [12]. With regard to cattle, the current publication is, to the author’s best knowledge, the second report of basophilic leukaemia [17].

Takahashi et al. [17] reported basophilic leukaemia in an eight-month-old calf suffering from anorexia and bloody stools. Similar to the present case, the spleen was enlarged on post-mortem examination, and histological examination revealed round tumour cells in various organs, including the spleen and the lymph nodes. Splenomegaly and enlarged lymph nodes are also typical symptoms in humans, in addition to bleeding tendency and susceptibility to infections [26, 27]. The final diagnosis in the case published by Takahashi et al. [17] was based on the presence of neoplastic cells in all examined organs together with their negative reactivity when stained for naphthol AS-D chloroacetate esterase [17]. Such staining of neoplastic cells was not performed in the present case. However, of the previously published case neither detailed information on clinical findings nor on collected blood nor bone marrow samples were available and therefore documented in the current case report. Comparing the two cases, the similarities regarding pathological results are striking with the additional details of blood and bone marrow in the present case. Detailed information about antigen characteristics of neoplastic cells could have been gained through immunohistochemistry, done regularly in small animal and human medicine. However, it was neither performed by Takahashi et al. [17] nor in the present case.

Anaemia, thrombocytopenia and neutropenia can be ascribed to depression of normal haematopoietic cells in the bone marrow. Due to the short residence time of granulocytes in peripheral blood and the short life span of thrombocytes (approximately 10 days), it seems reasonable that in the current case thrombocytopenia and neutropenia were severe [28]. Despite the relatively long lifespan of red blood cells in healthy calves (about 110–120 days), myelophthisis may affect the erythroid lineage of a growing animal with an expanding blood volume more severely than an adult being suffering from acute leukaemia. In addition, blood loss over multiple haemorrhages detected at necropsy may have contributed to moderate anaemia. Thrombocytopenia in the present case resulted in haemorrhagic diathesis as indicated clinically by prolonged bleeding at injection sites. The severity of leucocytosis during acute leukaemia is closely related to the release of leukaemic (blast) cells from bone marrow into peripheral blood and can thus be subject to considerable variation [29] as was also seen in the present case.

The calf exhibited signs of a neurological disorder. Massive cell infiltration into the vessels of the meninges may explain neurological involvement of the brain [5], reflecting apathy and tetraparesis/generalised ataxia. In the clinically suspected area of the last thoracic and first lumbar vertebrae, compression of the spinal cord caused by extramedullary bleeding (confirmed by MRI and necropsy) likely contributed to progressing signs of severe paresis of the hind limbs while the calf was hospitalised.

Generally, clinical signs may vary considerably in cases of acute leukaemia. In this case, pneumonia was suspected based on clinical symptoms and mild arterial hypoxia. Signs of a pulmonary disorder may have been caused by an increased susceptibility to infections [30] and/or massive infiltration of myeloid cells into the pulmonary vessels, thus impairing normal pulmonary function.

## Conclusions

In conclusion, despite its rarity, acute myeloid leukaemia with involvement of the basophilic lineage and acute basophilic leukaemia may be considered in calves with progressive deteriorating general condition, leucocytosis with moderate basophilic differentiation or haemorrhagic disorders.

## Abbreviations

AML: acute myeloid leukaemia
CSF: cerebrospinal fluid
EDTA: ethylenediaminetetraacetate
FLS: fat-liquor cerebrospinalis
MRI: magnetic resonance imaging
WHO: World Health Organisation
